# A cluster-randomised controlled trial of values-based training to promote autonomously held recovery values in mental health workers

**DOI:** 10.1186/s13012-015-0363-5

**Published:** 2016-02-02

**Authors:** Virginia Williams, Frank P. Deane, Lindsay G. Oades, Trevor P. Crowe, Joseph Ciarrochi, Retta Andresen

**Affiliations:** 1 School of Psychology, University of Wollongong, Wollongong, Australia; 2 Illawarra Institute for Mental Health, University of Wollongong, Wollongong, Australia; 3 Australian Institute of Business Wellbeing, University of Wollongong, Wollongong, Australia; 4 Institute of Positive Psychology and Education, Australian Catholic University, Sydney, Australia

**Keywords:** Values, Self-determination, Implementation, Mental health, Motivation

## Abstract

**Background:**

The implementation and use of evidence-based practices is a key priority for recovery-oriented mental health service provision. Training and development programmes for employees continue to be a key method of knowledge and skill development, despite acknowledged difficulties with uptake and maintenance of behaviour change. Self-determination theory suggests that autonomy, or a sense that behaviour is self-generated, is a key motivator to sustained behaviour change, in this case practices in mental health services. This study examined the utility of values-focused staff intervention as a specific, reproducible method of autonomy support.

**Methods:**

Mental health workers (*n* = 146) were assigned via cluster randomisation to either a values clarification condition or an active problem-solving control condition.

**Results:**

Results demonstrated that a structured values clarification exercise was useful in promoting integrated motivation for the changed practice and resulted in increased implementation planning.

**Conclusions:**

Structured values clarification intervention demonstrates utility as a reproducible means of autonomy support within the workplace. We discuss future directions for the study of autonomous motivation in the field of implementation science.

**Trial registration:**

ACTRN12613000353796

## Background

Implementation of evidence-based practice is a key priority of mental health service delivery [1, 2]. This priority arises out of a need to maximise efficiency within health systems and also out of a responsibility to provide efficacious services to mental health consumers in order to promote positive health outcomes. The challenge of translating research into practice is well acknowledged particularly in the mental health recovery field [3, 4]. Training and education programmes continue to be a primary approach to developing skills, knowledge and practices within workplace environments, including the mental health field. Training occurs despite limited uptake and maintenance of new, evidence-based methods of practice [5–7].

Previous research attempting to understand the barriers to uptake of newly learned work practices identifies factors ranging from organisational (e.g. lack of time, duplicitous paperwork, ill-equipped administrative and support systems) [8] and managerial issues (e.g. lack of management support and understanding of new practices, pressure for immediate results) [9] to individuals factors (e.g. employee skills, self-perceived competence to adopt new practices and motivation to adopt change) [10, 11].

Within mental health services, little objective support for the organisational, managerial and skill-related barriers cited most often by mental health workers as factors impeding transfer has been identified in previous research [12]. In contrast, lack of personal belief and commitment to the change appears to be a key practitioner barrier to implementation. Motivation for change has been identified as central to successful transfer and implementation in numerous studies e.g. [10, 11].

Motivation can be described as the force that energises an individual towards a specific goal or end-state [13, 14]. Whilst organisational factors continue to be a focus of workplace change studies, there is evidence to suggest that the degree of autonomous motivation for change at the level of individual staff has been somewhat neglected in organisational research [15]. Autonomous motivation can be described as the extent to which a perceived cause to action is experienced as self-determined, or regulated by oneself [16, 17]. Autonomy has been described as a basic human need [18] and predicts both purposeful striving towards desired end-states and continued performance in the face of adversity [19].

At the level of individual employees, wide-scale organisational development such as a change in work practices is likely to be experienced as imposed or externally regulated at least to some degree. The motivation to act has not been self-determined, or arisen from within the individual. Imposed change can create conditions of conformity and commitment to doing ‘what my employer says’, at the same time restricting an individual’s sense of autonomy and desire to personally express commitment to the change [20, 21]. The effects of imposed change have been widely studied in other contexts where communal needs (e.g. to comply with pro-social benchmarks or standards) at times reasonably restrict the individual’s right to autonomy and personal expression in order to promote the overall priorities of the group (e.g. [21–23]. Research over the past 20 years has led to the conclusion that individuals who experience their behaviour to be externally controlled, and motivated by a need to conform or keep an external party happy, are significantly less likely to spontaneously strive towards the set-standard, or to persist once the perceived controls cease [20, 23, 24].

This research has implications for how to best promote uptake of a newly learned, evidence-based practice. In most workplace settings, employees will be required to participate and complete tasks that relate to organisational priorities. The tasks are not self-selected, and at times, may be uninteresting. The challenge for change-agents and managers in organisations is similar to that faced by leaders in other contexts where there is a need for consistency and standardisation, that is, how is autonomy fostered for practices that are externally regulated, or ‘imposed’ upon individuals. Autonomy support has been described and researched extensively in educational and developmental contexts e.g. [17, 21, 25]. Autonomy support refers to practices that actively encourage initiative and provide a meaningful rationale for the task, in addition to minimising control and conformity-oriented language [19].

Autonomy supportive practices are thought to work by promoting the individual’s right to personal expression and facilitating internalisation of the values and approach being forwarded [21, 26]. In other words, rather than doing something because of pressure from somebody else (e.g. manager, supervisor) or to avoid an adverse consequence, an individual acts purposefully out of a sense that they wish to do so as the behaviour aligns with what they believe and value. To this end, the initially imposed practice or task is experienced as more self-determined, and autonomous motivation for striving is maximised. Autonomy support has been found to promote greater competence and mastery [27] higher performance [28] and higher achievement [29] when compared to other common approaches to motivating behavioural change (e.g. use of reward or punishment).

Autonomy support has been operationalised in terms of three elements: (1) acknowledging participant feelings, (2) offering a meaningful rationale for the task and (3) emphasising choice rather than using controlling language [30]. There is evidence to suggest that autonomy supportive practices are both teachable [22, 26] and that managerial influence is a significant factor in determining whether a workplace will be autonomous or control-oriented [31]. Autonomy support and related literature sit within an extensive body of research regarding Self-Determination Theory (SDT) [17, 19, 32]. However, concerns have been raised about the relative absence of applied SDT research in organisational contexts [16].

Whilst SDT and autonomy support are both validated within behaviour change and personality literatures, explication of how autonomy support looks in practice, beyond a set of general principles, is an area requiring further research. The need to identify empirically validated approaches to operationalising autonomy support in organisations is highlighted in [16].

The present study focuses on a values-focused training component that complements a 2-day employee development training intervention, as an example of a structured and reproducible autonomy supportive methodology.

### Values-based approaches to autonomy support

The process of internalisation has been indicated as the mechanism by which an imposed (or externally regulated) task or behaviour becomes more autonomously motivated (and self-regulated) [17, 19, 32, 33]. Internalisation as a construct has been figural within personality and behaviour-change literature over several decades and is understood to be an important adaptive and transformative process [34, 35]. Internalisation takes two forms according to the SDT and results in different types of behavioural regulation. SDT conceptualises motivation on a continuum, ranging from intrinsic and self-determined (autonomous) to extrinsic and externally regulated (controlled) at each end. Introjected and integrated motivation is between these poles, with the former being closer to extrinsic and the latter closer to intrinsic motivation [19, 32]. In a socially controlled environment such as the workplace, there is often limited scope for actual free choice and low frequency of tasks that are done for pure pleasure and enjoyment (intrinsic motivation). Autonomy supportive practices in socially controlled environments (e.g. workplaces) are therefore aiming to foster increased internalisation as evidenced by increased integrated motivation within individuals [35, 36].

One approach to the promotion of internalisation is to provide an opportunity for individual values to be clarified, discussed and validated, and then ‘matched’ against the values in which the externally driven change is embedded. To date, autonomy support has identified the need to validate individual feelings, offer a meaningful rationale, and minimise controlling language. A search of the BioMed Central database of journals using the terms ‘values’, ‘autonomy support’, ‘staff’ and ‘organisations’ in a variety of combinations returned no positive matches. To the best of our knowledge, the merit of targeted values-focused work as a way of operationalising autonomy support has not been investigated. This study investigates a structured, purposeful values-clarification intervention where personal values and workplace values are both explored and prioritised as an additional component to an evidence-based 2-day employee development training programme (Collaborative Recovery Model Training (CRMT)) [37]. Values have been identified as important predictors of behaviour [38, 39], whilst implementation plans have been highlighted as key to goal attainment [40]. As such, impacts of values clarification on plans to implement the new workplace practices will also be investigated as an early indicator of planned behaviour change.

We will explore these impacts in comparison to a control condition that will combine structured problem-solving and implementation planning with the CRMT programme. It is hypothesised that the mental health worker teams receiving the additional values-based training will show a greater increase in their integrated motivation for the new workplace practice following training than those in the control group teams. It is also expected that those receiving the values intervention will show a greater increase in plans to implement the new CRM practices following training than those in the control group. It is also useful to explore the impacts of this intervention on other forms of motivation that have been explicated in the SDT model as there is limited research at this component-level particularly in organisations [16]. Whilst intrinsic motivation is not expected to change, the potential changes to introjected and extrinsic motivation following a values-based intervention for staff are worthy of exploration.

## Methods

### Participants and procedures

Participants were 146 staff members recruited from four community-managed organisations that provide programmes to support individuals with severe and recurrent mental health challenges. Each organisation was a partner in an Australian Research Council grant project with the University (LP0990708). Using a computer-generated randomisation list, the research team randomly assigned mental health workers by work site to the experimental condition (values group) or the control condition (implementation group). Equal numbers of sites from within each partner organisation were randomly assigned to either the values or implementation group. Cluster randomisation was adopted due to the highly interdependent nature of mental health workers within workplaces and also to ensure fidelity to condition. For these reasons, it was not possible to blind participants to condition. All participants were aware of the alternate experimental condition, the hypotheses and perceived merits of each experimental group. Accredited trainers from the research team attended sites within each partner organisation and delivered the ‘standard’ training programme in addition to the appropriate condition-specific intervention. Responsibility for intervention delivery was maintained by the accredited trainers within the research team to promote fidelity to condition and integrity of intervention.

The standard component of the intervention involved delivery of the Collaborative Recovery Model training, which is an evidence-based staff development programme structured around six core principles or workplace values [41]. Participants assigned to the values group received a third day of training that comprised a structured values clarification card sorting process developed by Ciarrochi and Bailey [42]. The purpose of the task is to help individuals identify 15 principles or valued-directions that are most important to them from 60 values cards. The values were derived from the 10 universal values identified by Schwartz and colleagues, which have been validated in cross-cultural research [43, 44]. Example values include ‘Caring for others’ (derived from Benevolence value) and ‘showing respect for tradition’ (derived from Tradition value). The mental health workers were instructed through a three-stage sorting process in order to arrive at a set of 15 principles that represented the things most important to them in their life generally. Following this, a group discussion was facilitated around the following questions: ‘Is anyone willing to share what they found important? Is anyone surprised at how unimportant some principles were compared to others?’

The individuals in the values group were then guided through the values-clarification process again, but on this second occasion, they were asked to adopt a workplace focus. At the end of the three-step sort, each individual identified the 15 principles from within the 60 cards that mattered most to him or her at work. Following this, each individual was guided through a process that involved them evaluating whether they had a current desire to take action in relation to each principle (‘Do you want to put this principle into play?’) and their recent success in living each out (‘How successful have you been in living this value over the past 3 months’). This evaluative process was developed by Sheldon and colleagues and has been used extensively e.g. [20, 24]. To conclude this process, a group discussion was facilitated around the following questions: ‘Is anyone willing to share what they found important to them at work?’, ‘How much is there in common with life in general and the workplace?’, ‘Can you find ways to bring your life in general principles into your workplace?’

The mental health workers in the control condition (implementation group) also received a third day of training, instead focused on identifying organisational barriers and other challenges likely to exist in their workplaces as implementation of the newly acquired skills and practices occurred. They were also provided the opportunity to problem-solve the identified barriers under the facilitation of the university trainer. This process was structured around a ‘SWOT Analysis’ protocol (Strengths, Weaknesses, Opportunities, Threats) and is a methodology that has been used extensively in organisations [45].

The 3 days of intervention were run successively with data collected at the commencement of day 1 (time 1) and the completion of day 3 (time 2). All individuals who attended the training were given both written and verbal information clearly indicating that whilst participation in the training was part of their workplace requirements, participation in the research component was voluntary. Data collection and management was undertaken in accordance with conditions stipulated to and authorised by the Human Research Ethics Committee at the University (HE09/221). The training intervention was rolled out over an 11-month period across a total of 22 sites.

### Measures

#### Autonomous motivation

The measure of ‘autonomy’ for the six workplace principles that underpin the CRM was developed using the methodology devised by [24]. Respondents were asked to rate the extent to which controlled (extrinsic), introjected, integrated and intrinsic motivators contributed to their goal-directed efforts aligned with the newly trained work practices. Extrinsic motivation was measured by endorsement of the statement, ‘somebody else wants me to do it’; introjected motivation by, ‘I do this for approval or I will feel guilty if I don’t’; integrated motivation by, ‘I wholly endorse it as important’ and intrinsic motivation by, ‘I do this for fun and enjoyment’. The participants rated each item using a five-point Likert scale that ranged from ‘not at all for this reason’ to ‘entirely for this reason’. This measure has been used to understand value motivations in mental health workers previously e.g. [46]. Previous SDT research [20, 24] has used an aggregated autonomy score calculated by subtracting the total ‘controlled’ motivation for the specific workplace principle from the total ‘autonomous’ motivation for the same principle, such that Autonomy = (intrinsic + integrated) − (introjected + extrinsic). Recent research has identified potential limitations in this aggregated method, e.g. [23], and instead analysed each of the four motivations separately, e.g. [46]. Moreover, we were specifically interested in understanding changes in the different components of motivation identified by SDT, particularly integrated motivation. Thus, for each participant, a total of four motivation scores on each of the six underpinning CRM principles were attained both prior to intervention (time 1) and at the conclusion of condition-specific intervention (time 2).

#### Plans to implement

Participants were asked to rate the degree to which they were planning purposeful action aligned to CRM across its six principles. Using a five-point Likert scale, respondents indicated the extent to which they had made specific plans to implement the particular CRM principle, from ‘not at all’ to ‘very much so’. This methodology has been validated in previous research [47] and is compatible with the process utilised by [24].

#### Analyses

As indicated in the participant flow chart (Fig. 2), there was data loss due to attrition. Baseline checks for differences between those who completed data at time 2, and non-completers found no differences in demographic variables (e.g. age, gender, length of experience) or on experimental variables (e.g. T1 integrated motivation).

Repeated measures analysis of variance examined main and interaction effects for time (pre-training day 1 and post-training day 3) and condition (values versus implementation). Our analyses focused on those who completed measures at the different time points. A series of correlations between the four levels of autonomous motivation with plans to implement pre-training, and for pre-post training, changes in motivation and implementations plans were carried out to better understand the relationships between variables. Multiple regression analyses were conducted to determine the degree of variance in outcome variables related to motivation and plans to implement that was predicted by condition.

## Results

**Fig. 1:**
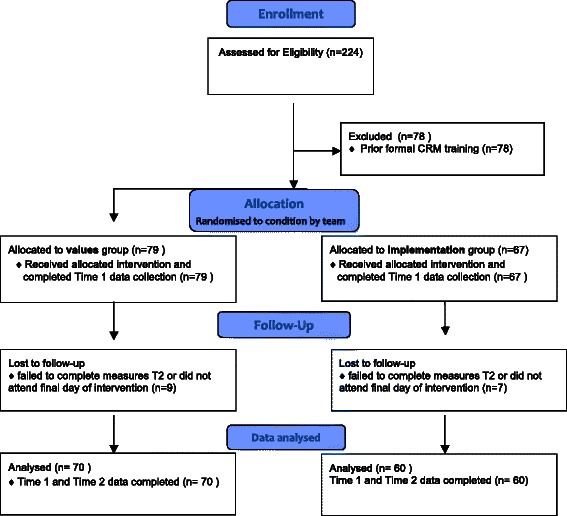
Consort Flow chart of participant recruitment and progression through intervention

Figure 1 indicates, a total of 146 participants were recruited, of whom 79 were randomised to the values condition and 67 to the implementation condition. Most participants were female (69 %). Participating mental health workers ranged in age from 18–60 and over, with 29 % aged 18–30, 27 % aged 31–40, 18 % aged 41–50 and 22 % aged 51–60 and 4 % were aged above 6 years. The modal period of service as a mental health worker was 1.5 years, with mean 4 years service. There were no significant differences between participants in each condition for baseline characteristics.

The CRM training intervention is based on a framework of mental health delivery that seeks to operationalise six core principles related to empowerment and actuation of personally meaningful goals. The central message in each of the six principles is outlined hereafter. Recovery involves and necessitates (1) a life that is meaningful to the individual, (2) collaborative relationships, (3) change enhancement, (4) strengths and values, (5) life visioning and goal setting, and (6) action planning and monitoring. Each principle embodies a core element embedded within the CRM, identified as foundational to mental health recovery. The knowledge, practices and skills trained within the standard CRMT programme are aligned with these principles.

Data relating to autonomous motivation and plans to implement was collected for each respondent in line with these six CRM principles. Exploratory analyses were conducted to better understand the co-relations between principles and between principles and outcome variables. Correlations and subsequent factor analyses revealed that the six core principles represented a unitary construct: All six principles were strongly correlated (*r*’s = . 54 to .74). Cronbach’s alpha for the six items was .88 indicating high consistency. This result was not unexpected as each of the principles is theoretically linked to the others as a key element of ‘recovery’. Further, the high intercorrelations serve to validate the evidence-based, conceptual model of mental health recovery that underpins the CRMT. To simplify further analyses, aggregated autonomy scores were utilised. Based on this, an overall score for CRM for each outcome variable (i.e. four motivations, plans to implement) at time 1 (pre) and time 2 (post) were calculated and used for subsequent analyses.

### Effect of condition

**Fig. 2:**
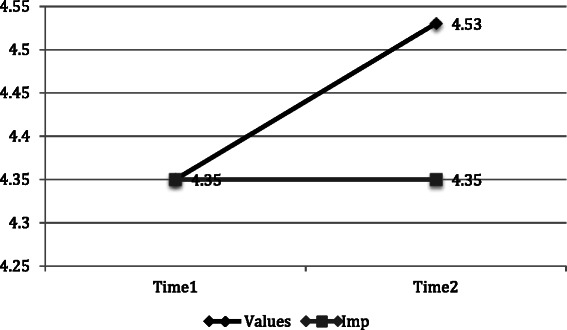
Significant interaction of time and condition on integrated motivation. F[1,129] = 6.67, p < .05

Repeated measures analysis of variance (ANOVA) for all participants who completed the intervention and provided data (T1 and T2) was conducted to examine the effect of time and condition on motivation and plans to implement training. No significant interactions were identified for extrinsic, introjected or intrinsic motivation. A significant positive time by condition interaction effect for integrated motivation was revealed, *F*[1,129] = 6.67, *p* < .05. Figure 2 depicts the significant interaction effect of time and condition on integrated motivation.

**Fig. 3:**
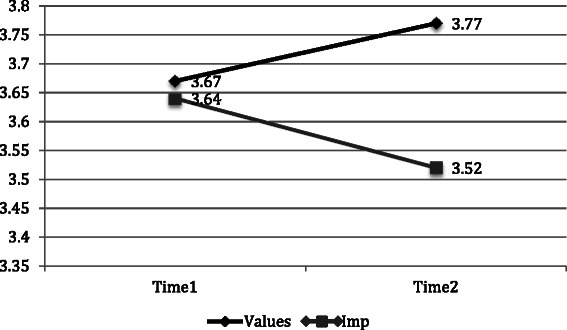
Significant interaction effect of time and condition on plans to implementation. F[1,129] = 4.80, p < .05

Repeated measures analysis of variance was conducted to examine the effect of time and condition on plans to implement the newly trained practice. Results for participants who completed the intervention (including data at T1 and T2) revealed a significant positive interaction for time and condition on plans to implement newly trained practices, with those in the values condition endorsing more highly than those in the implementation condition following intervention, *F*[1,129] = 4.80, *p* < .05. Figure 3 depicts the significant interaction effect of time and condition on plans to implement newly trained CRM practices.

Table 1 depicts all pre- and post-training means and standard errors for each motivation component and plans to implement the CRMT by condition.

**Table 1:** Means and standard error (pre and post) for intervention completers by condition

Variable	Condition	Time	Mean	St E	Number
Integrated motivation	Values	1	4.35	.06	70
		2	4.53	.06	70
	Implementation	1	4.35	.06	60
		2	4.35	.07	60
Intrinsic motivation	Values	1	3.42	.12	70
		2	3.32	.14	70
	Implementation	1	2.96	.13	60
		2	2.95	.15	60
Introjected motivation	Values	1	1.48	.09	70
		2	1.42	.10	70
	Implementation	1	1.53	.10	60
		2	1.50	.10	60
Extrinsic motivation	Values	1	1.48	.08	70
		2	1.34	.08	70
	Implementation	1	1.57	.09	60
		2	1.43	.09	60
Plans to implement	Values	1	3.67	.09	70
		2	3.77	.08	70
	Implementation	1	3.64	.09	60
		2	3.52	.09	60

### Correlational analyses

Correlations between the forms of motivation and plans to implement were conducted to better understand the relationships both prior to training and after intervention using Pearson correlation coefficient. At baseline, extrinsic motivation negatively correlated with plans to implement, *r* =−.21, *p* < . 05. Plans to implement were positively correlated with integrated motivation *r* = .49, *p* < . 01 and intrinsic motivation, *r* = .33, *p* < . 01. Change scores were calculated for each motivation component by subtracting T1 from T2, as were change scores for plans to implement. A significant relationship was found between change in plans to implement and change in integrated motivation (*r* = .26, *p* < . 01). Additionally, there was a significant negative relationship between change in introjected motivation and change in plans to implement (*r* =−.26, *p* < . 01); change in extrinsic motivation was also negatively correlated with change in plans to implement (*r* =−.20, *p* < . 05). The findings are summarised in Table 2 below.

**Table 2:** Interrelations between plans to implement and motivation type at time 1 and for pre- to post changes

Correlations between plans to implement and motivation at time 1 (pre-training)		Correlations between pre- to post changes in plans to implement and motivation	
	Plans to implement		Change in plans to implement
	(*n* = 144)		(*n* = 130)
Motivation type		Motivation type	
Extrinsic	−.21*	Extrinsic	−.20*
Introjected	−.16	Introjected	−.26**
Integrated	.49**	Integrated	.26**
Intrinsic	.33**	Intrinsic	−.02 NS

*NS* not significant (*p* > .05)

*Significant, *p* < .05; **Significant, *p* < .01

Regression analyses were conducted to further explore the relationships described in Table 2. A hierarchical regression was conducted with variables entered stepwise based on previous research and the strength of the interrelations we identified, with integrated motivation entered in step 1, intrinsic motivation at step 2, introjected motivation at step 3 and finally extrinsic motivation at step 4. The model was set with *p* value at .05. Controlling for baseline plans to implement, integrated motivation was the only variable that uniquely predicted plans to implement at time 2. Results are presented in Table 3.

**Table 3:** Time 1 motivation types predicting variance in plans to implement at time 2

	*B*	Std. error	Beta
Step 1			
Plans to implement (T2)	1.91	.51	
Integrated	.41	.11	.26**
Step 2			
Plans to implement (T2)	1.88	.51	
Integrated	.38	.12	.25**
Intrinsic	.04	.06	.05
Step 3			
Plans to implement (T2)	2.04	.55	
Integrated	.37	.12	.23*
Intrinsic	.04	.06	.06
Introjected	−.05	.06	−.06
Step 4			
Plans to implement (T2)	1.99	.56	
Integrated	.38	.12	.24*
Intrinsic	.04	.06	.05
Introjected	−.08	.09	−.10
Extrinsic	.04	.09	−.05

Note: *R* squared = .07 for step 1, change *R* squared = .00 for step 2, .00 for step 3, .00 for step 4

* significant, p <.05, ** significant, p <.01

## Discussion

Development of employee skills within the mental health field is challenging. Workplace training programmes continue to be a prime method of organisational change, despite somewhat disappointing impacts on implementation in general and specifically in the mental health field. Enhancement of employee autonomous motivation to change is an area of inquiry that has received relatively little empirical attention [16]. In particular, the identification of structured, reproducible approaches to supporting worker autonomy for change has been highlighted as a specific need. Within socially controlled environments such as the workplace, integrated motivation represents the optimal level of internalisation of an otherwise imposed behavioural regulation [35]. The use of a structured values clarification process as an intervention to follow training in a new set of evidence-based mental health recovery practices was tested for its applicability as a means of supporting integrated motivation for change.

Aligned with our main hypothesis, a significant increase in integrated motivation for a newly trained work practice was found for staff that participated in a structured values clarification intervention compared to those who participated in structured problem-solving. These results lend support for values clarification as a means to promoting employee internalisation of an otherwise imposed workplace change. Additionally, staff in the values condition also evidenced a significant increase in plans to implement to the workplace initiative compared to those in the implementation (problem-solving) condition. Implementation planning is associated with increased purposeful goal attainment and striving [48]. This suggests enabling staff to identify and clarify personal and workplace values embedded within a newly trained workplace initiative may lead to increased personal ownership and planned transfer.

We envisage this kind of intervention, as an adjunct to standard knowledge and skills training, would have utility in any context where transfer of training is a specific concern or target. Gaining the buy in from staff is anecdotally acknowledged as an important factor in bringing about behaviour change, though receives less research attention than other workplace initiatives like bonuses, rewards and opportunities [49].

These findings indicate that it is possible to provide a brief, reproducible intervention that enables staff to identify and work with ‘intangibles’ such as their personally meaningful values and beliefs, and such  an intervention can have positive effects on motivation for change.

The results did not indicate significant effects for aggregated autonomous motivation (i.e. integrated + intrinsic − introjected + controlled), which aligns with the contemporary SDT research [25] and also fits with expectations of motivation for change in a controlled environment, such as the workplace. Furthermore, there was no significant effect of condition on intrinsic motivation (e.g. I do this for fun or enjoyment), introjected motivation (e.g. I do this because I would feel guilty otherwise) or controlled motivation (e.g. do this because somebody else wants me to) when they were reviewed separately. This finding suggests that future work centred on promoting autonomy and uptake in controlled environments may do well to focus on integrated motivation specifically as a means to bringing about internalisation and self-directed implementation of the new practice. It also adds to the increasing understanding about the motivation continuum explicated within SDT.

Correlation analyses between motivation types and plans to implement were conducted to better understand the relationship between motivation and plans to implement across the intervention period (Table 3). Increases in integrated motivation from time 1 to time 2 were positively correlated with increases in plans to implement the new workplace practice from pre to post. Regression analyses indicated integrated motivation at time 1 uniquely predicted plans to implement at time 2 for our sample when compared with the other forms of motivation. These findings further suggest that integrated motivation is a construct highly relevant to implementation planning and worthy of further research as a mechanism of bringing about workplace change.

### Limitations and future directions

Our research investigates changes in motivation and planning following a brief intervention, across a period of 3 days. Whilst the results are positive, the improvements in motivation and planning are anticipatory and may not lead to changed practice or sustained uptake. Moreover, research relating to values has indicated personal value systems to be a stable construct, changing relatively little over time [38, 44]. Longitudinal research acknowledging the relatively stable nature of the values construct and allowing investigation of changes to ongoing implementation is highlighted as a need within the field of organisational behaviour change broadly and specifically in mental health recovery.

Data loss due to attrition was an issue in this project and is acknowledged as a practical and statistical concern for applied research generally [50]. Comparison of pre-training variables for mental health workers who completed all elements of the intervention (i.e. pre-data collection, 2 days standard training, day 3 of condition-specific intervention and post-training data) with those who did not complete all elements indicate that there were no differences in baseline data (e.g. demographics) or on pre-training experimental variables (e.g. integrated motivation). This intervention did not focus on the pre-training organisational context or in any way seek to actively increase the extent to which the training was experienced as ‘owned’ by those who participated. For example, assessing for and understanding readiness for change, allowing individuals to have a say in some elements of the training (even if this is practical in nature) or eliciting some pre-training discussion about the individual’s experience of their workplace may help to reduce the sense that the new practices were ‘forced upon’ and increase involvement in the change. Readiness for change, and understanding the pre-change environment, seems to represent a step towards the creation of an autonomy supportive work climate and is well supported in behaviour change research [51]. Talking *about* change prior to it happening may actually undermine the extent to which it is perceived as forced or imposed, thereby aligning with key priorities identified by [16].

In terms of operationalising autonomy support and enhancing its relevance to organisational contexts, our research has emphasised the second element of three identified underpinning components, namely providing a meaningful rationale for the change. The values-clarification intervention facilitated awareness and clarification of personal and work values but did not go so far as to elicit and explore the affective responses of staff to the change process itself (component 1 of autonomy support). The third component (minimising controlling language and emphasising choice) was arguably targeted in the values-clarification process, but consideration of a more transparent discussion about implementation may be warranted in future applications.

Further interventions may do well to build in a structured opportunity for staff to identify and express feelings related to the workplace change and to talk directly about the how, why and when of implementing the newly learned skills. This would represent a morphing of our two interventions to some degree (i.e. allowing some implementation planning and problem-solving as per the control group) but with continued and primary emphasis on allowing mental health workers to internalise the imposed change through identifying the alignment with deeply held values and beliefs. This may lead to further positive impacts on internalisation of the values embedded within a workplace change over and above the significant findings realised in this study. Given the relevance of values concordant goal-setting and striving to personal wellbeing [20, 24], the present research also highlights the need to better understand possible dual impacts of value-based interventions on effective goal striving and employee well-being in an era where organisational effectiveness and responsibility to personnel are increasingly emphasised [52, 53].

## Conclusions

This research has implications for mental health services and other organisations wishing to promote transfer of workplace change through staff training programmes. The results indicate that provision of opportunity for staff to identify and clarify personal and work values after training in the to-be-adopted practices positively impacted the extent to which individuals wholly endorsed and internalised the practice as well as their plans to implement the change.

This study has highlighted integrated motivation as important to change and the potential for structured values intervention as an explicit approach to autonomy support in socially controlled environments, such as the workplace. We believe this study provides promising indication that such intervention can be both replicable and brief and still have a positive impact on the degree to individual workers ‘buy in’ to an otherwise imposed workplace change. From this perspective, this study adds to the current knowledge and application of SDT as a theory of work motivation and identifies a brief and relatively cost-effective method that potentially enhances the uptake of evidence into practice. The current findings are relevant to any context where the research-practice gap pervades. Further work is required to determine the relevance of values intervention on employee motivation for change in the longer term, in addition to transfer and maintenance of skills and practice.
